# Supplementary material for: Thermodynamic architecture and conformational plasticity of GPCRs
Source: Nat Commun. 2023 Jan 9;14:128. doi: 10.1038/s41467-023-35790-z (PMC9829892; doi:10.1038/s41467-023-35790-z)
Supplement: Supplementary file 1 — Supplementary Information [file 41467_2023_35790_MOESM1_ESM.pdf]

Supplementary Information

Thermodynamic Architecture and Conformational Plasticity of GPCRs

*Sathvik Anantakrishnan & Athi N. Naganathan**

Department of Biotechnology, Bhupat & Jyoti Mehta School of Biosciences, Indian Institute of Technology Madras, Chennai 600036, India.

*e-mail: athi@iitm.ac.in

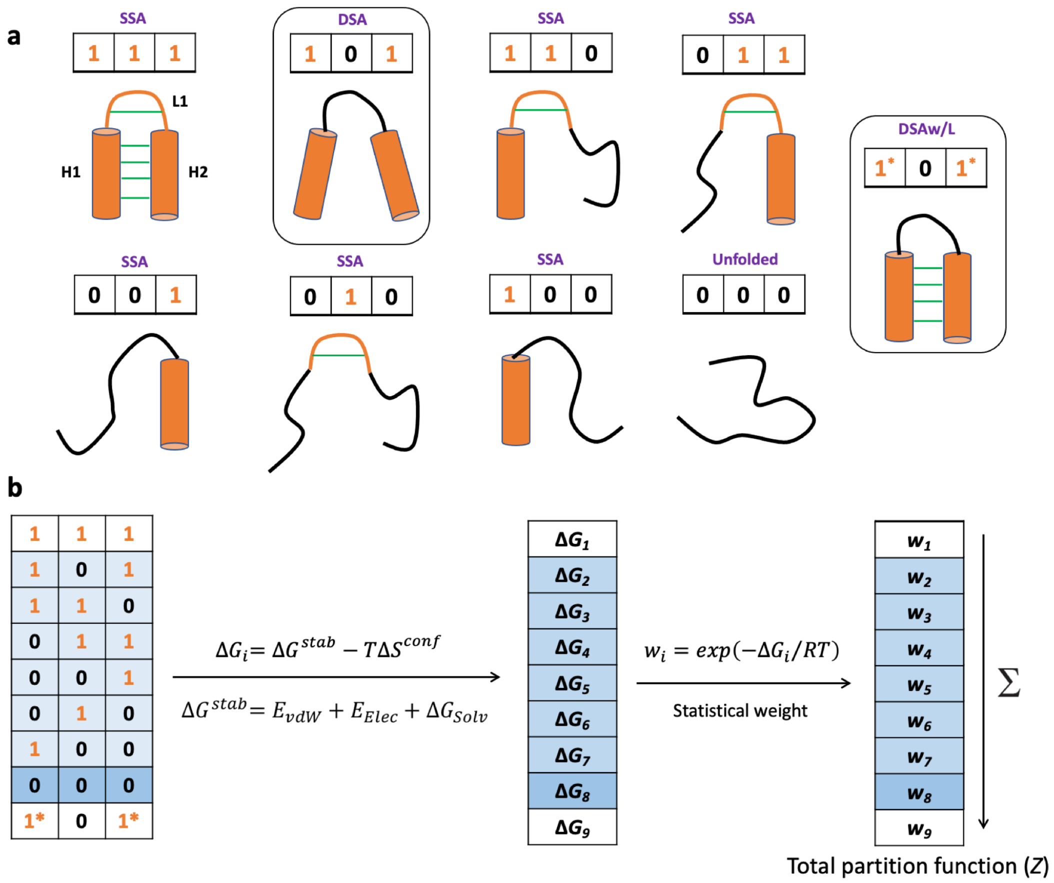

Supplementary Fig. 1 Microstate definitions in the WSME model. (**a**) WSME model microstates for a highly coarse-grained version of a protein where the *entire* secondary structure elements (H1, L1, and H2) are considered as blocks. *1* represents the folded block and *0* represented the unfolded block. For such a three-block representation, there can be a maximum of 6 SSA states (single sequence approximation), one defined by DSA (double sequence approximation), one DSAw/L state (DSA with loop) and one fully unfolded state. In DSA (boxed and adjacent to the fully folded protein), the helices H1 and H2 do not interact as L1 is unfolded, while in DSAw/L the two helices can interact despite the unfolded L1 (the state to the extreme right of the panel; asterisks are employed to distinguish this state from DSA). The interactions within the secondary structure elements are also considered. *In the current work, we employ a similar formalism for GPCRs but coarse-grain the protein in four-residue blocks, resulting in millions of such conformational substates (Table S1).* (**b**) Since the structure of the each of the microstates are known (from the PDB file), the corresponding free-energy of microstates (ΔG_i) can be calculated considering the balance between stabilization free energy (ΔG^{stab}) and conformational entropy (ΔS^{conf}). The former includes contributions from van der Waals interactions (vdW), electrostatics (Elec) and solvation (empirical solvation free-energy term; see main text). From the free energies, the statistical weights and hence the total partition function can be calculated (summation of statistical weights). The partition function can, in turn, be employed to calculate heat capacity profiles, free-energy profiles and free-energy landscapes (see main text).

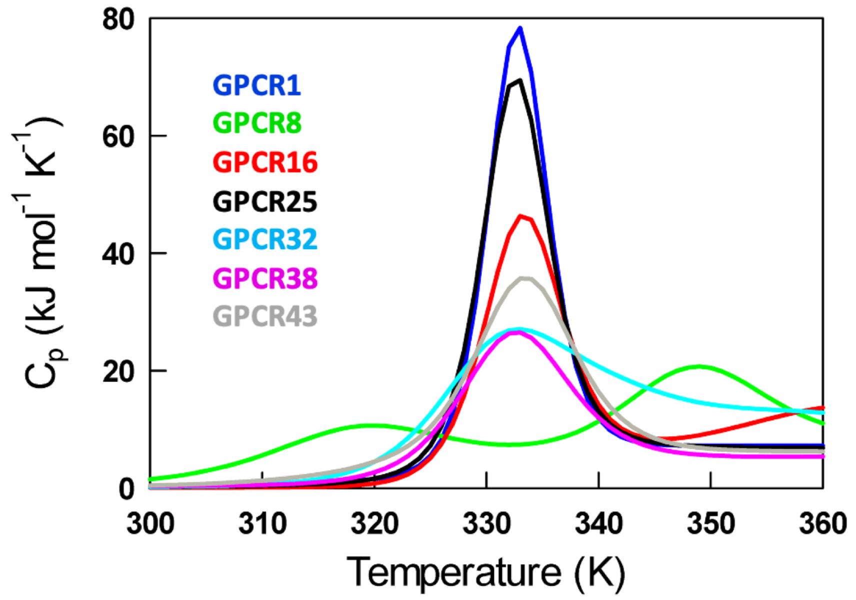

Supplementary Fig. 2 Predicted heat capacity profiles of representative GPCRs that span the spectrum of thermodynamic unfolding behaviors. See Table S1 for the name and PDB ids of GPCRs. Source data are provided as a Source Data file.

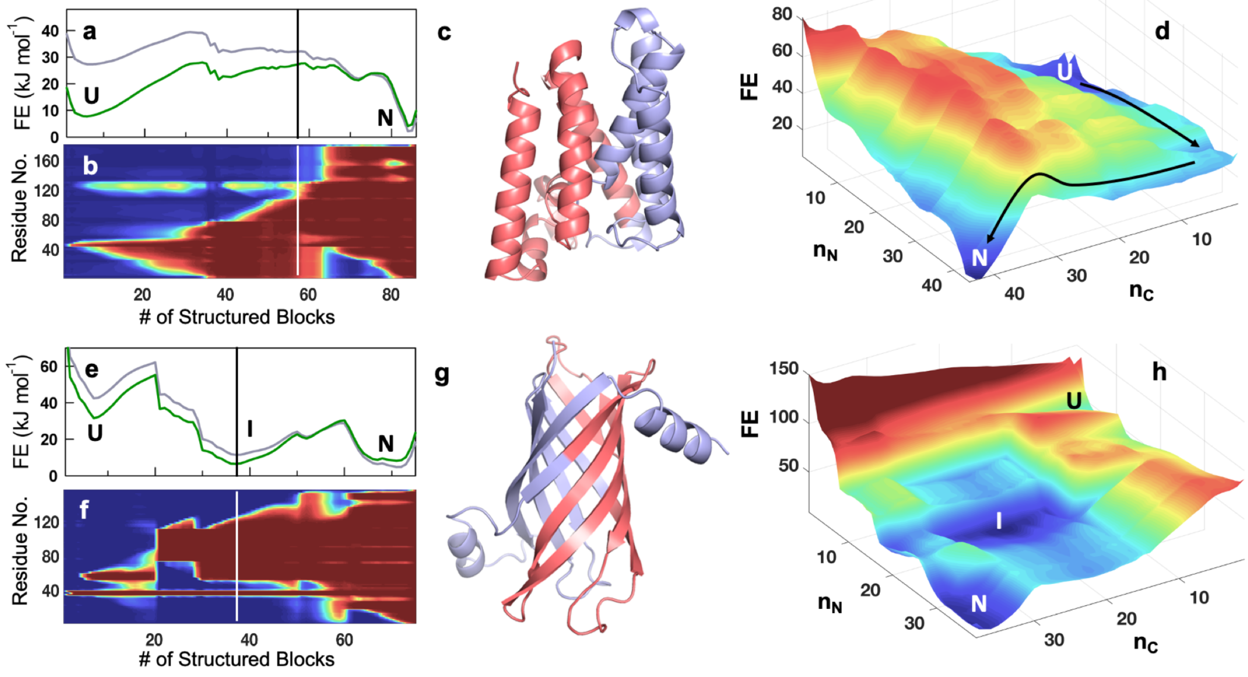

Supplementary Fig. 3 (**a**-**b**) Conformational landscape of the helical membrane protein GlpG. Panel **a** plots the free energy profile at 310 K and the melting temperature of 333 K (grey and green, respectively) as a function of the reaction coordinate, the number of structured blocks. Panel **b** plots the folding probability of different protein regions (y-axis) as a function of the reaction coordinate (x-axis) in the spectral scale (blue represents zero folding probability while red represents a folding probability of one). At 57 structured blocks (the vertical dashed in panels **a** and **b**), the N-terminal half is more folded than the C-terminal half. Protein regions that are folded and unfolded at 57 structured blocks are mapped onto the structure in salmon and light blue, respectively (panel **c**). A similar observation on the folding mechanism can be made from the perspective of the two-dimensional folding landscape as a function of the number of folded structured blocks in the N- and C-terminal halves (n_N and n_C, respectively; panel **d**). The vertical axis is the free energy (FE) in kJ mol^{-1}. The arrows point to the direction of folding starting from the unfolded state (U) to the folded state (N). It can be seen that the N-terminal region folds first followed by the C-terminal region. Note that these inferences are made from equilibrium populations and not from kinetic modeling that can potentially reveal higher complexity. (**e**-**h**) Folding mechanism of the beta-sheet-rich PagP following the same color code as panels **a**-**d**. In panel **f**, it can be seen that the C-terminal half is relatively more structured compared to the N-terminal half; this is mapped onto the structure in panel **g**. In PagP, the salmon colored region in panel **g** forms an intermediate (I). The intermediate can also be observed in the 2D landscape (panel **h**). Source data are provided as a Source Data file.

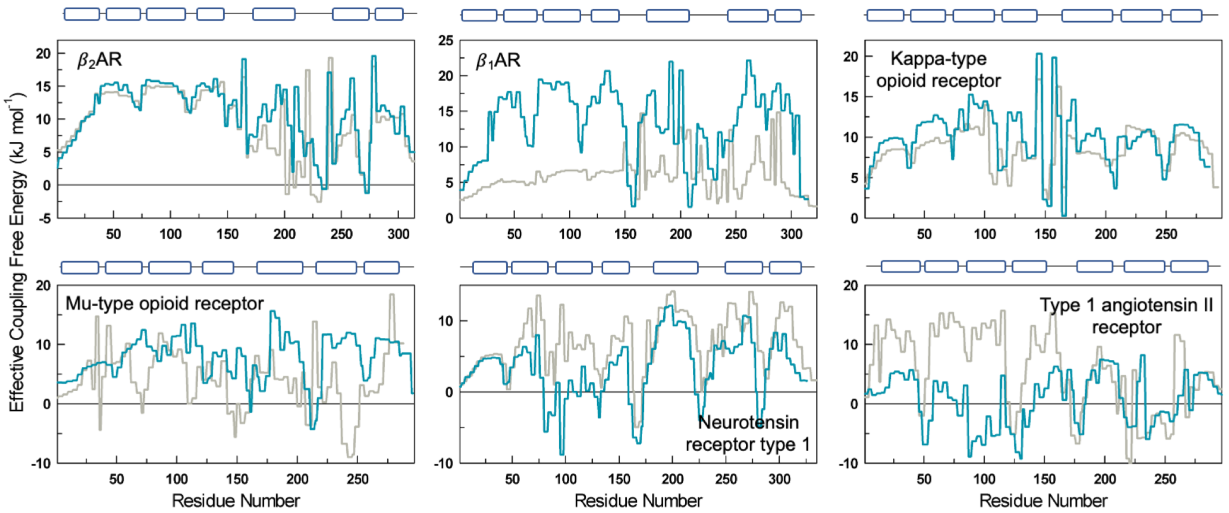

Supplementary Fig. 4 Effective coupling energies for the inactive (gray) and active (dark cyan) forms of the GPCRs. The cartoon on top displays the length and position of the transmembrane helices. Also refer to Fig. 6 in the main text and the associated discussion. Source data are provided as a Source Data file.

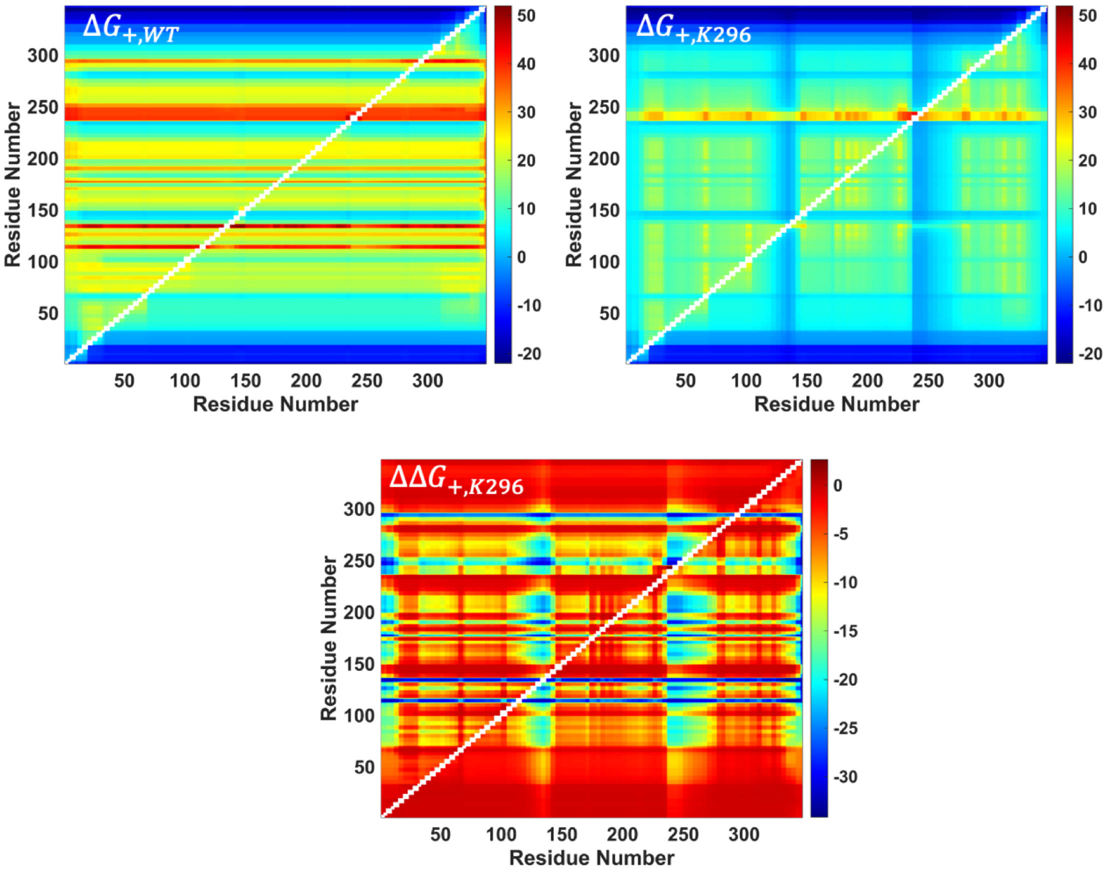

Supplementary Fig. 5 The positive coupling matrix for WT Rhodopsin (top left), the mutant K296 (top right) and the difference between the two (bottom row). It can be seen that the residues in the mutant are less coupled than the WT (top right). Source data are provided as a Source Data file.

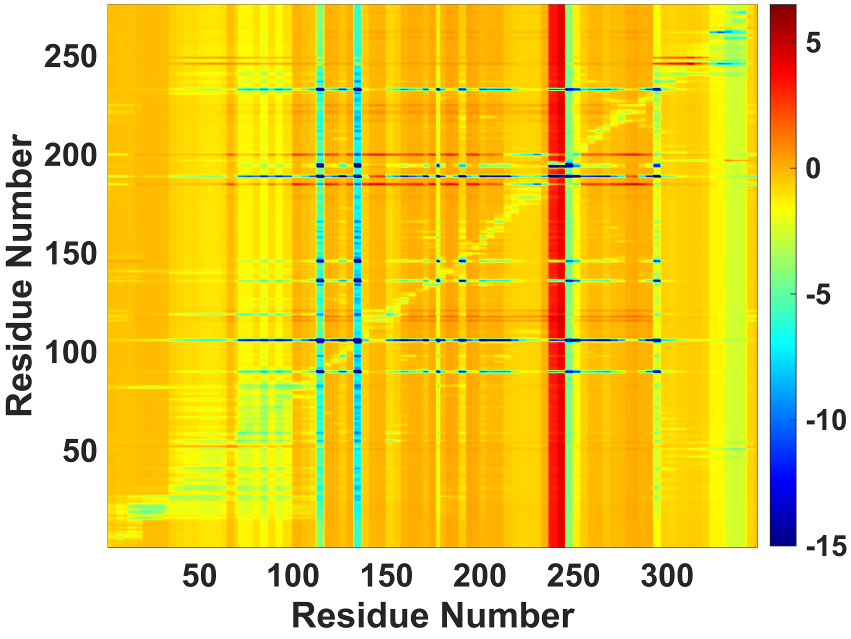

Supplementary Fig. 6 Mutational response matrix - every row in this matrix represents the $\langle\Delta\Delta G_{+}\rangle$ across all residues for a specific alanine mutation or perturbation in Rhodopsin. The average and standard deviation across the rows is shown as Fig. **7b** and **7c** in the main text. The lowest $\langle\Delta\Delta G_{+}\rangle$ is -47 kJ mol^{-1}, but the color range at the lower end of the spectrum is constrained here to -15 kJ mol^{-1} for the sake of clarity. Source data are provided as a Source Data file.

Supplementary Table 1 Database of inactive GPCR structures.

GPCR Index	Protein	PDB ID	UniProt ID	Class	No. of Residues (*N*)	No. of Blocks (N_b)	No. of Microstates
**1**	Rhodopsin	1U19	P02699	A	349	90	5,349,436
**2**	C-X-C chemokine receptor type 1	2LNL	P25024	A	296	77	2,855,854
**3**	Beta-2 adrenergic receptor	2RH1	P07550	A	314	84	4,053,141
**4**	C-X-C chemokine receptor type 4	3ODU	P61073	A	293	75	2,568,801
**5**	Sphingosine 1-phosphate receptor 1	3V2Y	P21453	A	311	82	3,678,644
**6**	Proteinase-activated receptor 1	3VW7	P25116	A	288	77	2,855,854
**7**	Beta-1 adrenergic receptor (Turkey)	4BVN	P07700	A	323	86	4,455,532
**8**	Kappa-type opioid receptor	4DJH	P41145	A	293	76	2,709,477
**9**	Mu-type opioid receptor (Mouse)	4DKL	P42866	A	288	76	2,709,477
**10**	Delta-type opioid receptor	4N6H	P41143	A	303	78	3,008,084
**11**	Metabotropic glutamate receptor 1	4OR2	Q13255	C	263	69	1,836,206
**12**	P2Y purinoceptor 12	4PXZ	Q9H244	A	291	75	2,568,801
**13**	Neurotensin receptor type 1	4XES	P20789	A	335	90	5,349,436
**14**	P2Y purinoceptor 1	4XNV	P47900	A	296	76	2,709,477
**15**	G-protein coupled receptor homolog US28 [Human cytomegalovirus (strain AD169) (HHV-5) (Human herpesvirus 5)]	4XT1	P69332	Viral	296	79	3,166,321
**16**	Type-1 angiotensin II receptor	4YAY	P30556	A	306	79	3,166,321
**17**	Lysophosphatidic acid receptor 1	4Z35	Q92633	A	304	81	3,501,442
**18**	Orexin receptor type 1	4ZJ8	O43613	A	348	92	5,843,749
**19**	Nociceptin receptor	5DHG	P41146	A	285	74	2,433,676

**20**	C-C chemokine receptor type 9	5LWE	P51686	A	298	76	2,709,477
**21**	Proteinase-activated receptor 2	5NDD	P55085	A	301	78	3,008,084
**22**	Free fatty acid receptor 1	5TZR	O14842	A	283	73	2,303,954
**23**	Adenosine receptor A1	5UEN	P30542	A	308	82	3,678,644
**24**	C-C chemokine receptor type 5	5UIW	P51681	A	301	79	3,166,321
**25**	Type-2 angiotensin II receptor	5UNF	P50052	A	292	74	2,433,676
**26**	Apelin receptor	5VBL	P35414	A	312	78	3,008,084
**27**	Glucagon-like peptide 1 receptor	5VEW	P43220		287	77	2,855,854
**28**	Neuropeptide Y receptor type 1	5ZBQ	P25929	A	320	85	4,250,766
**29**	Platelet-activating factor receptor	5ZKP	P25105	A	310	80	3,330,721
**30**	Cannabinoid receptor 2	5ZTY	P34972	A	299	76	2,709,477
**31**	5-hydroxytryptamine receptor 2A	6A94	P28223	A	330	87	4,667,609
**32**	Frizzled-4	6BD4	Q9ULV1	F	333	89	5,114,386
**33**	C5a anaphylatoxin chemotactic receptor 1	6C1R	P21730	A	298	78	3,008,084
**34**	Prostaglandin D2 receptor 2	6D27	Q9Y5Y4	A	323	83	3,862,489
**35**	Metabotropic glutamate receptor 5	6FFI	P41594	C	264	68	1,731,349
**36**	C-C chemokine receptor type 2	6GPX	P41597	A	286	75	2,568,801
**37**	Substance-P receptor	6HLP	P25103	A	301	77	2,855,854
**38**	Endothelin receptor type B	6IGK	P24530	A	318	82	3,678,644
**39**	Thromboxane A2 receptor	6IIU	P21731	A	314	84	4,053,141
**40**	Prostaglandin E2 receptor EP3 subtype	6M9T	P43115	A	308	82	3,678,644
**41**	Melatonin receptor type 1A	6ME2	P48039	A	299	79	3,166,321
**42**	Melatonin receptor type 1B	6ME6	P49286	A	294	76	2,709,477

**43**	Calcitonin receptor	6NIY	P30988	B	277	74	2,433,676
**44**	C-C chemokine receptor type 7	6QZH	P32248	A	290	73	2,303,954
**45**	Cysteinyl leukotriene receptor 2	6RZ6	Q9NS75	A	294	75	2,568,801

Supplementary Table 2 Model parameters and thermodynamic outputs.

GPCR Index	Protein	PDB ID	vdW Interaction Energy (J mol^{-1})	Strongly Coupled Residues at 310 K (f_c in %)	No. of Intermediates from 1D FE Profile at T_m (1*RT* threshold)
**1**	Rhodopsin	1U19	-48.2	9.8	1
**2**	C-X-C chemokine receptor type 1	2LNL	-56.3	9.4	0
**3**	Beta-2 adrenergic receptor	2RH1	-46.7	15.6	2
**4**	C-X-C chemokine receptor type 4	3ODU	-52.0	23.9	0
**5**	Sphingosine 1-phosphate receptor 1	3V2Y	-49.2	7.4	3
**6**	Proteinase-activated receptor 1	3VW7	-52.3	16.7	0
**7**	Beta-1 adrenergic receptor (Turkey)	4BVN	-46.7	11.1	1
**8**	Kappa-type opioid receptor	4DJH	-47.4	7.5	2
**9**	Mu-type opioid receptor (Mouse)	4DKL	-49.9	12.2	0
**10**	Delta-type opioid receptor	4N6H	-46.1	19.1	2
**11**	Metabotropic glutamate receptor 1	4OR2	-48.6	10.6	1
**12**	P2Y purinoceptor 12	4PXZ	-47.6	21.0	2
**13**	Neurotensin receptor type 1	4XES	-53.2	20.3	1
**14**	P2Y purinoceptor 1	4XNV	-46.6	12.2	6
**15**	G-protein coupled receptor homolog US28 [Human cytomegalovirus (strain AD169) (HHV-5) (Human herpesvirus 5)]	4XT1	-48.3	7.1	3
**16**	Type-1 angiotensin II receptor	4YAY	-50.1	21.6	0
**17**	Lysophosphatidic acid receptor	4Z35	-51.9	8.9	0

	1				
**18**	Orexin receptor type 1	4ZJ8	-49.70	10.3	6
**19**	Nociceptin receptor	5DHG	-54.4	8.4	2
**20**	C-C chemokine receptor type 9	5LWE	-45.2	15.1	1
**21**	Proteinase-activated receptor 2	5NDD	-46.0	13.0	0
**22**	Free fatty acid receptor 1	5TZR	-52.4	15.2	0
**23**	Adenosine receptor A1	5UEN	-47.3	10.1	2
**24**	C-C chemokine receptor type 5	5UIW	-45.6	9.6	0
**25**	Type-2 angiotensin II receptor	5UNF	-50.2	11.3	2
**26**	Apelin receptor	5VBL	-50.2	15.7	1
**27**	Glucagon-like peptide 1 receptor	5VEW	-47.9	13.2	4
**28**	Neuropeptide Y receptor type 1	5ZBQ	-48.5	20.0	1
**29**	Platelet-activating factor receptor	5ZKP	-48.3	18.4	2
**30**	Cannabinoid receptor 2	5ZTY	-47.3	14.7	1
**31**	5-hydroxytryptamine receptor 2A	6A94	-48.2	16.1	0
**32**	Frizzled-4	6BD4	-45.7	9.6	1
**33**	C5a anaphylatoxin chemotactic receptor 1	6C1R	-47.8	7.0	4
**34**	Prostaglandin D2 receptor 2	6D27	-52.3	16.7	3
**35**	Metabotropic glutamate receptor 5	6FFI	-49.8	15.2	0
**36**	C-C chemokine receptor type 2	6GPX	-47.7	16.8	2
**37**	Substance-P receptor	6HLP	-48.7	12.3	3
**38**	Endothelin receptor type B	6IGK	-45.2	11.3	1
**39**	Thromboxane A2 receptor	6IIU	-49.3	10.2	3

**40**	Prostaglandin E2 receptor EP3 subtype	6M9T	-56.0	3.9	3
**41**	Melatonin receptor type 1A	6ME2	-48.1	9.4	1
**42**	Melatonin receptor type 1B	6ME6	-46.8	9.5	1
**43**	Calcitonin receptor	6NIY	-48.0	13.0	1
**44**	C-C chemokine receptor type 7	6QZH	-45.7	12.8	5
**45**	Cysteinyl leukotriene receptor 2	6RZ6	-47.8	10.9	0

Supplementary Table 3 Model parameters for GlpG and PagP.

Protein	GlpG	PagP
PDB ID	2XOV	1THQ
No. of Residues	181	157
Block size	2	2
No. of Blocks	86	75
No. of Microstates	4,455,532	2,568,801
vdW Interaction Energy (J mol^{-1})	-49.0	-46.5

Supplementary Table 4 Model parameters employed for predicting free energy profiles from active and inactive structures at 310 K. (note that the parameters for inactive structures are same in Supplementary Table 2)

GPCR Index	Inactive Structures			Active Structures		
	PDB ID	Resolution (Å)	vdW Interaction Energy (J mol^{-1})	PDB ID	Resolution (Å)	vdW Interaction Energy (J mol^{-1})
**1**	1U19	2.2	-48.2	5W0P	3.0	-47.8
**3**	2RH1	2.4	-46.7	4LDE	2.8	-48.7
**7**	4BVN	2.1	-46.7	6H7N	2.5	-50.3
**8**	4DJH	2.9	-47.4	6B73	3.1	-49.5
**9**	4DKL	2.8	-49.9	5C1M	2.1	-49.6
**13**	4XES	2.6	-53.2	6OS9	3.0	-55.0
**16**	4YAY	2.9	-50.1	6DO1	2.9	-53.1
**23**	5UEN	3.2	-47.3	6D9H	3.6	-48.9
