# Supplementary material for: Thermodynamic architecture and conformational plasticity of GPCRs
Source: Nat Commun. 2023 Jan 9;14:128. doi: 10.1038/s41467-023-35790-z (PMC9829892; doi:10.1038/s41467-023-35790-z)
Supplement: Supplementary file 2 — Reporting Summary [file 41467_2023_35790_MOESM2_ESM.pdf]

nature portfolio

Corresponding author(s): Athi N Naganathan

Last updated by author(s): Dec 13, 2022

# Reporting Summary

Nature Portfolio wishes to improve the reproducibility of the work that we publish. This form provides structure for consistency and transparency in reporting. For further information on Nature Portfolio policies, see our Editorial Policies and the Editorial Policy Checklist.

## Statistics

For all statistical analyses, confirm that the following items are present in the figure legend, table legend, main text, or Methods section.

| n/a | Confirmed | |
|---|---|---|
| ☐ | ☒ | The exact sample size (*n*) for each experimental group/condition, given as a discrete number and unit of measurement |
| ☒ | ☐ | A statement on whether measurements were taken from distinct samples or whether the same sample was measured repeatedly |
| ☒ | ☐ | The statistical test(s) used AND whether they are one- or two-sided<br>*Only common tests should be described solely by name; describe more complex techniques in the Methods section.* |
| ☒ | ☐ | A description of all covariates tested |
| ☒ | ☐ | A description of any assumptions or corrections, such as tests of normality and adjustment for multiple comparisons |
| ☐ | ☒ | A full description of the statistical parameters including central tendency (e.g. means) or other basic estimates (e.g. regression coefficient) AND variation (e.g. standard deviation) or associated estimates of uncertainty (e.g. confidence intervals) |
| ☒ | ☐ | For null hypothesis testing, the test statistic (e.g. *F*, *t*, *r*) with confidence intervals, effect sizes, degrees of freedom and *P* value noted<br>*Give P values as exact values whenever suitable.* |
| ☒ | ☐ | For Bayesian analysis, information on the choice of priors and Markov chain Monte Carlo settings |
| ☒ | ☐ | For hierarchical and complex designs, identification of the appropriate level for tests and full reporting of outcomes |
| ☒ | ☐ | Estimates of effect sizes (e.g. Cohen's *d*, Pearson's *r*), indicating how they were calculated |

*Our web collection on statistics for biologists contains articles on many of the points above.*

## Software and code

Policy information about availability of computer code

| | |
|---|---|
| Data collection | Matlab 2020a was used to run the simulations. |
| Data analysis | Matlab 2020a and PyMol was used to analyze the outputs. Codes are available at https://github.com/AthiNaganathan/GPCR-Landscapes |

For manuscripts utilizing custom algorithms or software that are central to the research but not yet described in published literature, software must be made available to editors and reviewers. We strongly encourage code deposition in a community repository (e.g. GitHub). See the Nature Portfolio guidelines for submitting code & software for further information.

## Data

Policy information about availability of data

All manuscripts must include a data availability statement. This statement should provide the following information, where applicable:
- Accession codes, unique identifiers, or web links for publicly available datasets
- A description of any restrictions on data availability
- For clinical datasets or third party data, please ensure that the statement adheres to our policy

All data are available in Github repository https://github.com/AthiNaganathan/GPCR-Landscapes. GPCR-EXP database (https://zhanggroup.org/GPCR-EXP) was employed to download high-resolution GPCR structures.

# Human research participants

Policy information about studies involving human research participants and Sex and Gender in Research.

| | |
|---|---|
| Reporting on sex and gender | Not applicable. |
| Population characteristics | Not applicable. |
| Recruitment | Not applicable. |
| Ethics oversight | Not applicable. |

Note that full information on the approval of the study protocol must also be provided in the manuscript.

# Field-specific reporting

Please select the one below that is the best fit for your research. If you are not sure, read the appropriate sections before making your selection.

[x] Life sciences [ ] Behavioural & social sciences [ ] Ecological, evolutionary & environmental sciences

For a reference copy of the document with all sections, see nature.com/documents/nr-reporting-summary-flat.pdf

# Life sciences study design

All studies must disclose on these points even when the disclosure is negative.

| | |
|---|---|
| Sample size | GPCR structures from the GPCR-EXP database were employed. 67 high-resolution structures were originally downloaded out of which 45 are used in the current study. |
| Data exclusions | GPCR structures with large extracellular loops or domains were exlcuded (22 out of 67 were excluded) as they cannot be modeled by a single dielectric constant. Please see the 'Methods' section for additional details. |
| Replication | All the codes have been run to verify the reproducibility of results. |
| Randomization | The current work is a computational analysis employing a statistical mechanical model and hence randomization is not applicable. |
| Blinding | The model employed takes in protein structure as input to provide detailed predictions and hence blinding is not applicable. |

# Reporting for specific materials, systems and methods

We require information from authors about some types of materials, experimental systems and methods used in many studies. Here, indicate whether each material, system or method listed is relevant to your study. If you are not sure if a list item applies to your research, read the appropriate section before selecting a response.

## Materials & experimental systems

| n/a | Involved in the study | |
|---|---|---|
| [x] | [ ] | Antibodies |
| [x] | [ ] | Eukaryotic cell lines |
| [x] | [ ] | Palaeontology and archaeology |
| [x] | [ ] | Animals and other organisms |
| [x] | [ ] | Clinical data |
| [x] | [ ] | Dual use research of concern |

## Methods

| n/a | Involved in the study | |
|---|---|---|
| [x] | [ ] | ChIP-seq |
| [x] | [ ] | Flow cytometry |
| [x] | [ ] | MRI-based neuroimaging |
